# Skills-Based Human Capital Management in Latvian University Hospitals: A Qualitative Multi-Institutional Study

**DOI:** 10.3390/healthcare14142032

**Published:** 2026-07-08

**Authors:** Evita Grigoroviča, Andreta Slavinska, Guntis Bahs, Tatjana Muravska, Edgars Edelmers

**Affiliations:** 1 Medical Education Technology Centre, Rīga Stradiņš University, Anninmuizas Boulevard 26a, LV-1067 Riga, Latvia; evita.grigorovica@rsu.lv (E.G.); andreta.slavinska@rsu.lv (A.S.); 2 Department of Internal Diseases, Rīga Stradiņš University, Dzirciema Street 16, LV-1007 Riga, Latvia; guntis.bahs@rsu.lv; 3 Faculty of Social Sciences, Rīga Stradiņš University, Dzirciema Street 16, LV-1007 Riga, Latvia; tatjana.muravska@rsu.lv; 4 Research Centre, Rīga Stradiņš University, Konsula Street 21, LV-1007 Riga, Latvia

**Keywords:** skills-based human capital management, physician skills, human capital, competency framework, entrustable professional activities, skills portfolio, healthcare workforce, healthcare management, patient safety, physician safety, qualitative research, reflexive thematic analysis, Latvia, health policy

## Abstract

Background and Objectives: Health systems increasingly require workforce governance approaches that move beyond formal qualifications and make physician skills more visible for planning, education, quality management, and resource allocation. However, evidence remains limited on how physician skill information is organisationally documented and used within hospitals, particularly in small health systems. This study examined how information on physician skills is organisationally documented, interpreted, and used for workforce governance across all Latvian university hospitals, and identified organisational, cultural, and digital barriers to systematic skills monitoring. Materials and Methods: A qualitative, exploratory, multi-institutional design was adopted. Semi-structured interviews were conducted with 12 stakeholders from human resources, education, quality, and finance across all three Latvian university hospitals. Data were analysed using reflexive thematic analysis, with reporting guided by COREQ and SRQR. Results: Eight interrelated themes were identified across three domains. Current practices were fragmented and primarily focused on formal qualifications rather than verifiable practical skills. Internally delivered education was partially documented, whereas externally acquired skills remained largely outside organisational systems. Skill gaps were usually identified reactively through incidents, complaints, or managerial observation. Training needs were determined through decentralised channels, and skill assessment depended heavily on local leadership judgement. Participants also highlighted financial blind spots, weak digital infrastructure, governance ambiguity, cultural resistance, and limited staffing capacity as barriers to implementation. At the same time, respondents consistently reported that improved skill visibility could support strategic workforce planning, targeted education, patient safety, better financial justification, and greater organisational transparency. Conclusions: Latvian university hospitals appear to recognise the organisational value of physician skill visibility but currently lack integrated systems for capturing and using such information systematically. The findings support the need for a context-sensitive, phased approach to skills-based workforce governance and inform a data-informed conceptual framework (Three-Layer Skills Governance Model) for aligning regulatory expectations, organisational processes, and individual skill records in small health systems.

## 1. Introduction

International guidance increasingly emphasises that health systems must optimise service delivery, strengthen workforce planning, and improve data infrastructures to support better decisions about staff numbers, locations, allocation, and capability development. The World Health Organization (WHO) Framework for Action on the Health and Care Workforce in the European Region (2023–2030) prioritises education and skills, team redesign, digital solutions, comprehensive workforce policies, and improved data [1], while the Global Strategy on Human Resources for Health advocates competency-based reforms and stronger workforce information systems [2]. At the European Union level, the 2024 Council Conclusions on the Future of the European Health Union call on Member States to address workforce shortages through investment, knowledge sharing, and digital tools [3], and *Health at a Glance: Europe 2024* frames workforce shortages and healthy longevity as interconnected priorities [4]. These policy priorities are particularly important in a context of wider demographic, technological, and socioeconomic change, where patient expectations are rising, populations are ageing, health spending is increasing in response to increasingly complex needs, and fiscal pressures constrain resource allocation [5].

In healthcare, workforce challenges are not only quantitative but also qualitative. Clinical quality and patient safety depend not only on the number of professionals employed, but also on whether practitioners possess and can apply the skills required for safe care. The WHO estimates that approximately one in ten patients is harmed in healthcare, with a substantial proportion of this harm being preventable [6]. OECD analyses similarly show that preventable harm absorbs a considerable share of healthcare resources and that failures in safety are often associated with deficits in skills, coordination, and team functioning [7]. From this perspective, skills and competencies are not merely matters of individual professional development; they are organisational and system-level assets that affect service reliability, quality improvement, patient safety, physician safety, and resource use. Yet in many settings, workforce information systems remain oriented toward staffing numbers, service activity, and expenditure rather than toward the practical capabilities available to organisations in real time [7].

Latvia faces these structural pressures under tighter fiscal and demographic constraints. *Latvia: Country Health Profile 2023* identifies chronic workforce shortages and pronounced staff ageing [8], while the *Health Workforce Strategy in Latvia* recommends building an integrated national database of health professionals, improving workforce planning models, and developing life-cycle pathways for skills enhancement [9]. The Latvian *Public Health Guidelines 2021–2027* similarly identify human-resource provision, competency development, and stronger data use as priority action areas, explicitly noting that weak data use in human resource management constrains evidence-based decision-making [10]. Taken together, these policy documents suggest that the challenge is not only workforce scarcity, but also the limited visibility and governability of workforce skills and competencies. In small health systems, where specialised expertise is concentrated in a small number of tertiary institutions, fragmented skills and competency information may have consequences not only for individual organisations but also for the adaptability of the system as a whole [4,8,9,10].

Despite increasing international emphasis on skills-based workforce governance, evidence remains limited on how physician skill information is documented, interpreted, and used within hospitals as part of organisational workforce governance. Existing research has predominantly focused on educational settings, individual professional development, or broader workforce policy and planning [11,12], leaving organisational skills-governance mechanisms underexplored. This gap is especially relevant in small European health systems, where tertiary-level capacity is concentrated in a limited number of university hospitals and fragmented data systems may constrain broader workforce planning [4,8,9]. Accordingly, this study examines how information on physician skills is organisationally documented, interpreted, and used for workforce governance across all Latvian university hospitals, from the perspectives of human resources, education, quality, and finance stakeholders. It further identifies organisational, cultural, and digital barriers to systematic skills monitoring and seeks to inform the development of a more structured approach to skills-based workforce governance in comparable settings.

## 2. Literature Review and Theoretical Framework

### 2.1. Human Capital Theory and the Shift Toward Skills-Based Human Capital Management

The present study is grounded in a skills-based human capital perspective. Human Capital Theory conceptualises the knowledge, skills, and abilities of individuals as critical for both personal productivity and organisational performance [13,14,15]. In healthcare, human capital encompasses the health status, education experience, professional knowledge, skills, and competencies of healthcare workers, all of which are essential for delivering quality care and achieving health-system goals. Evidence indicates that human capital management (HCM) is associated with improved organisational performance [11,16], employee well-being [17,18,19], and patient outcomes [17,20]. Strategic investments in training, engagement, and workforce planning therefore enable healthcare organisations to deliver high-quality care more sustainably [11,21].

At the same time, contemporary health workforce research suggests that possessing human capital is not the same as organisationally mobilising it. A growing body of work in health workforce and healthcare management research therefore shifts the analytical focus from qualifications as static credentials toward the organisational processes by which skills and competencies are developed, recognised, deployed, and aligned with service needs [11,22]. This shift is central to strategic human resource management because workforce value depends not only on who is employed, but also on whether the organisation can identify what staff are capable of doing, where gaps exist, and how performance can be improved over time [11,22]. Building on this foundation, skills-based HCM has emerged as a distinct evolution of strategic HRM that treats verifiable competencies—rather than job titles or formal credentials—as the unit of analysis for workforce planning, deployment, and development [23,24].

This emphasis on skills as the operational foundation of physician performance aligns closely with Human Capital Theory, which conceptualises knowledge, skills, and abilities as core productive assets contributing to organisational performance and long-term system outcomes [13,25]. In healthcare, this perspective is particularly salient, as human resources are widely recognised as a key determinant of healthcare quality and population health outcomes [22]. Consequently, HCM extends beyond quantitative workforce planning to include the strategic development, deployment, and utilisation of workforce capabilities in alignment with organisational and system needs. Contemporary health workforce frameworks emphasise that workforce performance is shaped not only by staffing levels, but also by how effectively skills are distributed, combined, and applied within healthcare delivery processes [11,22,26]. From this perspective, the systematic identification and management of skills become central to organisational performance and service quality.

### 2.2. Competencies, Professional Capability, and Entrustable Professional Activities as Analytical Lenses

The shift toward skills-based HCM broadens the analytical lens through which healthcare workforce capacity can be understood. It enables workforce capacity to be examined in terms of competencies, professional capability, and Entrustable Professional Activities (EPAs), each of which captures a distinct but interrelated dimension of professional performance. These constructs should therefore be understood not as competing concepts, but as complementary analytical lenses within a multi-layered framework of healthcare workforce analysis.

Within this framework, competency is typically understood as the ability to perform professional tasks through the application of knowledge, skills, and attitudes, operationalised as observable and assessable performance within defined standards of practice. In the medical education literature, competencies have been described as “multi-domain clinical expertise, comprising medical knowledge, skills, attitudes, and metacognitive capabilities” inferred from performance evaluations of trainees [27]. Other definitions emphasise competency as the ability to execute a task or action with the necessary knowledge [28]. In this sense, competencies refer to the structured and assessable qualities of professionals and provide an important basis for defining expected standards of performance. Competencies are behavioural in nature, in that they concern the integration of knowledge, skills, and attitudes in the performance of tasks within a given context, and they are expected to be durable, trainable, and measurable [29].

By contrast, professional capability captures a broader and more adaptive dimension of professional performance. While competencies describe what a professional is expected to know and do, capability reflects the integrative and context-sensitive use of these competencies in complex, evolving, and uncertain environments. Professional capability is commonly defined as the ability to apply knowledge, skills, and personal attributes effectively across both predictable and uncertain contexts, with a strong emphasis on adaptability, learning, and performance in complex situations [30,31,32]. Capable individuals do not merely possess specialised skills; they also have the confidence and judgement to employ them in varied circumstances and to continue expanding their knowledge and skills over time [30]. Capability therefore includes not only knowing how to perform, but also knowing how to learn, adapt, and creatively integrate prior knowledge, skills, judgement, and experience in both new and familiar situations [31].

EPAs, in turn, operationalise this integration by translating competencies into observable units of professional practice. EPAs are defined as observable and measurable units of professional work that represent core clinical responsibilities and can be entrusted to healthcare professionals once sufficient competence has been demonstrated [33,34,35,36]. They are independently executable, observable, and measurable in both process and outcome, making them suitable for entrustment decisions [34]. The essential distinction is that competencies describe characteristics of individuals, whereas EPAs describe the work that must be done. EPAs therefore require the integration of multiple competencies and are more directly tied to clinical practice and patient care [36]. Collectively, these analytical lenses imply that physician professionalism is not defined through a single construct, but through a layered architecture of competencies, capabilities, and practice-based activities that together inform education, assessment, and workforce governance.

### 2.3. International Frameworks and National Approaches to Physician Professionalism

These analytical distinctions are reflected in international practice. National systems vary in how they formulate physician professionalism requirements: competency-based frameworks remain dominant, but capability-oriented language and EPA-based operationalisation are increasingly used in parallel. For example, Canada combines the CanMEDS competency framework with Competence by Design, in which discipline-specific standards include both competencies and EPAs [37,38]. The United States has operationalised expectations for graduates through the AAMC Core EPAs for Entering Residency [39], whereas the Netherlands has implemented EPAs nationally across postgraduate specialty programmes as part of a broader competency-based reform [40]. In the United Kingdom, regulatory standards for medical graduates are framed primarily through knowledge, skills, behaviours, and capabilities [41,42], while in Australia EPAs have been incorporated into the national prevocational training framework as descriptions of essential work undertaken by junior doctors [43,44].

At the global level, the WHO Global Competency Framework for Universal Health Coverage defines a set of core knowledge, skills, and attitudes required for health and care workers to deliver high-quality, people-centred services. The framework is designed to support curriculum development and align education with the evolving demands of healthcare practice, using an “adapt and adopt” approach to ensure contextual relevance. By establishing a shared language of competence focused on service delivery rather than professional titles, it enhances accountability between education and employment and enables more flexible, system-oriented workforce planning [29].

Notwithstanding the diversity of analytical lenses and national approaches to defining and regulating physician professionalism, a unifying element across these frameworks is the central role of physicians’ skills. In practice, it is the repertoire of skills that underpins the formation of competencies, shapes professional capability, and ultimately enables the performance of EPAs in real clinical contexts. As such, skills constitute a foundational precondition for assessing whether existing frameworks and professional standards are meaningfully achieved in practice. This places increasing importance on systematic approaches to capturing and organising information on physician skills, as robust and accessible skill-related data provide a critical basis for evaluating workforce capacity, identifying gaps, and supporting evidence-informed decision-making at organisational and system levels.

### 2.4. Skills Data, Hospital Governance, and Workforce Analytics

The ability to generate, integrate, and use structured information on physician skills is a critical enabler of effective workforce governance. Evidence from health systems research demonstrates that reliable workforce data—including skill mix, distribution, and capacity—are essential for evidence-informed planning, performance management, and policy development [45,46]. Data-driven approaches to healthcare management further show that integrating workforce-related information into organisational analytics can improve quality outcomes and resource utilisation, enabling organisations to identify performance gaps and optimise care delivery processes [47]. Building on this foundation, emerging approaches to workforce management increasingly adopt a skills-based human capital perspective, in which skills—rather than job titles or formal credentials—serve as the primary unit of analysis for understanding workforce capacity and guiding decision-making. This shift is consistent with broader developments in health workforce policy and management, which emphasise the need to align workforce capabilities with changing patient needs, technological advancements, and system-level priorities through more flexible, data-informed models of workforce planning and deployment [11,22,46,48].

The relevance of a skills-based perspective is particularly evident in hospital settings. Hospitals operate in complex, interdependent environments characterised by technological change, interdisciplinary coordination, and continuing pressure to demonstrate quality, safety, and efficiency. At the same time, they are investing in data infrastructures and analytics to improve operational and strategic decision-making. Health analytics literature distinguishes descriptive, diagnostic, predictive, prescriptive, and discovery functions that can support management across operational and strategic levels [49], and empirical studies show that integrated data use can improve hospital quality performance and reveal substantial room for improvement across mortality, readmission, and length-of-stay indicators [47]. However, hospital data systems are typically organised around activity, finance, and outcomes rather than workforce skills, competencies, capabilities, or EPAs. As a result, organisations often lack systematic information on what skill-related resources are available, how they are distributed, and how gaps relate to service needs and patient safety.

This challenge is especially pronounced in university hospitals, which combine specialised care with education, supervision, and workforce development. Their dual institutional role makes skills visibility relevant not only for patient care, but also for training quality, staffing allocation, supervision capacity, and career progression. Studies of multi-institutional EPA implementation demonstrate that such approaches can support structured progression, shared expectations, and organisational learning, but their effectiveness depends on institutional commitment, implementation support, and alignment with local routines [50,51]. These findings highlight that frameworks alone are insufficient; their organisational value depends on whether they are embedded in governance arrangements, information systems, and institutional culture [50,51,52]. Research on human capital management in hospital settings similarly indicates that workforce performance is shaped by organisational context, leadership, communication, and support structures, not merely by formal qualifications. Systematic reviews show that leadership behaviours, autonomy, organisational support, and workplace relationships influence staff performance and engagement [53,54].

A further limitation concerns workforce analytics, skills mapping, and the measurement of continuous development. Strategic workforce analysis studies demonstrate that skills gaps can be identified and used to guide targeted interventions [55], while reviews of lifelong learning measurement in healthcare reveal substantial inconsistency in the way capability development is conceptualised and operationalised [12]. This fragmentation limits organisations’ ability to generate comparable, longitudinal data on skills and to use such information for workforce governance. In this sense, the challenge is not only to educate competent professionals, but also to build organisational systems capable of rendering skills visible and actionable.

### 2.5. Resource-Based and Systems Perspectives on Skills Governance

The Resource-Based View (RBV) provides an additional lens for understanding why physician skills matter strategically in healthcare organisations. RBV argues that differences in organisational performance are shaped by internal resources and capabilities rather than by external conditions alone, and contemporary RBV literature has explicitly linked this logic to human resources and human capital as key sources of value creation [56]. In healthcare settings, resources include both tangible assets—such as buildings, equipment, technologies, and financial resources—and intangible assets such as knowledge, skills, routines, and organisational culture. Healthcare strategic management research suggests that, while tangible resources remain essential for service delivery, their performance contribution depends on how effectively they are activated through organisational capabilities and human expertise [57,58]. This distinction is particularly relevant in a skills-based human capital context, because physician skills are not simply individual attributes; they are intangible strategic resources whose value emerges when they are coordinated with tangible resources and embedded in organisational processes of care delivery, innovation, and improvement [56,59].

A systems perspective further strengthens this analytical framing by recognising that healthcare is not a collection of isolated professional roles or organisational functions, but a complex adaptive system composed of interconnected people, processes, technologies, and institutions. In such systems, performance depends not only on the quality of individual elements, but also on the relationships and interactions between them, meaning that workforce capability must be understood in relation to wider organisational structures, information flows, and patterns of coordination [60,61]. Systems thinking in health research similarly emphasises interdependence, dynamic behaviour, feedback, and the identification of leverage points, suggesting that improvements in workforce governance require attention not only to professional standards themselves, but also to the system conditions through which they are enacted, monitored, and sustained [61,62]. From this perspective, physician skills are not simply individual attributes; they are embedded within organisational systems of assessment, deployment, learning, and decision-making, and their value depends on whether these systems are able to capture and use skill-related information effectively.

The growing literature on resilience in healthcare reinforces the importance of skills visibility. Resilience has been conceptualised as the capacity of healthcare systems and organisations to adapt, maintain function, learn, and improve under changing conditions [63]. Recent hospital resilience research shows that effective adaptation depends not only on structural resources and staffing levels, but also on learning capacity, communication, interdisciplinary coordination, and the ability to mobilise expertise under pressure [64,65]. Yet the literature provides limited guidance on how hospitals can systematically capture and use information on workforce skills and competencies to support such resilience in routine governance.

### 2.6. Theoretical Framework, Research Gap, and Study Contribution

Taken together, the literature reviewed above supports a multi-layered theoretical framework for analysing physician skills governance in hospitals. Human Capital Theory explains why physician knowledge, skills, and abilities represent productive organisational assets [13,14,15]. Skills-based HCM extends this logic by shifting attention from formal credentials to verifiable skills as actionable units for workforce planning, development, and deployment [11,22,23,24]. Competency, capability, and EPA frameworks provide complementary analytical lenses for understanding how professional performance can be defined, assessed, and translated into observable work [27,28,29,30,31,32,33,34,35,36]. RBV further conceptualises physician skills as strategic intangible resources whose value depends on organisational deployment and complementarity with tangible resources [56,57,58,59]. Systems thinking emphasises that skills become organisationally meaningful only when embedded in processes, information flows, feedback mechanisms, and governance structures [60,61,62].

Despite these advances, a critical gap remains in how healthcare organisations systematically capture, structure, and use data on physician skills. Existing research has predominantly focused on educational settings, individual professional development, or broader workforce policy and planning [11,12], leaving organisational skills-governance mechanisms underexplored. While existing frameworks define competencies, capabilities, and professional activities, they provide limited guidance on how skill-related information is operationalised within hospital processes or integrated into workforce governance. In practice, data on physician skills are often fragmented across educational records, certification systems, local administrative processes, and individual professional portfolios, limiting their usability for strategic decision-making and workforce planning.

The effective implementation of a skills-based human capital approach therefore presupposes a clear understanding of the existing organisational landscape. This includes identifying current practices and processes through which physician skills are documented and utilised, as well as recognising gaps, inconsistencies, and missing links in the institutional data and governance architecture. Such a diagnostic perspective is essential to ensure that future skills-based approaches are not introduced as abstract models, but are grounded in real organisational conditions and capable of building on existing practices.

The present study addresses this gap by examining how information on physician skills is organisationally documented, interpreted, and used for workforce governance across all Latvian university hospitals. By focusing on human resources, education, quality, and finance stakeholders, the study analyses how skill-related information is distributed across organisational functions and how it may be better integrated into skills-based workforce governance. In doing so, the study contributes empirical insight into organisational approaches to physician skills governance in a small health system context and informs a data-informed conceptual Three-Layer Skills Governance framework for aligning regulatory expectations, hospital-level processes, and individual skill records.

## 3. Materials and Methods

### 3.1. Aim and Study Design

This study aimed to examine how information on physician skills is organisationally documented, interpreted, and used for workforce governance across all Latvian university hospitals, from the perspectives of human resources, education, quality, and finance stakeholders, and to identify organisational, cultural, and digital barriers to systematic skills monitoring.

Rather than assessing individual physician competencies or performance, the study was designed as an organisational-level investigation of skills governance processes, focusing on how skill-related information is embedded within institutional structures, decision-making practices, and data systems. To operationalise this aim, the study pursued the following objectives:

1. To describe current organisational practices for documenting and managing information on physician skills across Latvian university hospitals;
2. To analyse how skill-related information is interpreted and used by different organisational functions (human resources, education, quality, and finance) in workforce governance;
3. To identify organisational, cultural, and digital barriers that limit the development of systematic skills monitoring approaches;
4. To explore how skill-related information is linked to workforce planning, training prioritisation, patient safety processes, and financial decision-making.

A qualitative, exploratory, multi-institutional design was adopted to capture in-depth organisational perspectives on skills governance across different functional domains within hospitals. This design is particularly suitable for examining complex, context-dependent processes—such as the documentation, interpretation, and use of skills data—which are shaped by organisational structures, professional roles, and institutional practices that cannot be fully captured through quantitative methods alone.

The multi-institutional approach enabled comparison across organisations operating within the same national health system, thereby providing insight into both shared and context-specific practices. The study was conducted and reported in accordance with the Consolidated Criteria for Reporting Qualitative Research (COREQ) [66] and the Standards for Reporting Qualitative Research (SRQR) [67].

### 3.2. Setting and Participants

The study was conducted across all Latvian university hospitals, which represent the highest level of specialised and academic healthcare provision in the country. These institutions play a central role in clinical service delivery, medical education, and professional skills and competency development, making them a critical setting for examining skills-based workforce governance.

Hospitals in Latvia that provide state-funded healthcare in a 24-h inpatient setting (Level V medical institutions) include Pauls Stradiņš Clinical University Hospital, Riga Eastern Clinical University Hospital, and Children’s Clinical University Hospital. All three institutions were included in the study to ensure comprehensive coverage of the national university hospital sector.

Purposive maximum-variation sampling was applied to capture perspectives from key organisational functions involved in skills governance. From each hospital, four participants were recruited—one from each of the following domains: (i) human resources, (ii) education, (iii) quality management, and (iv) finance. This resulted in a structured 4 × 3 sampling matrix and a total of twelve participants (n = 12). All participants held managerial or coordinating responsibilities directly related to physician skill or competency management within their organisational unit. This sampling strategy was chosen to ensure that the study captured diverse but complementary organisational perspectives on how skill-related information is generated, interpreted, and used across different governance domains.

### 3.3. Data Collection

Semi-structured interviews were used to balance thematic consistency with sufficient flexibility, allowing participants to describe institutional practices from the perspective of their functional role while enabling comparative analysis across cases.

A total of twelve interviews were conducted in Latvian via the Microsoft Teams platform between June 2025 and March 2026. Interviews lasted between 30 and 60 min (median 47 min). All interviews were conducted by the first author (E.G., female, MSc), who has prior training in qualitative research methods. The interviewer had no prior personal or professional relationship with participants. Participants were informed about the study purpose, the researcher’s institutional affiliation, and the focus on skills-based workforce governance prior to providing informed consent.

All interviews were video-recorded with participant consent and transcribed verbatim. Field notes were maintained to document contextual observations, emerging analytical insights, and reflections during data collection.

The interview guide was developed based on the study aim and conceptual framing and was pilot-tested with one informant external to the participating institutions. Minor refinements were made prior to formal data collection. The guide included five thematic domains: (i) current practices in documenting physician competencies and skills; (ii) identification and assessment of skill gaps; (iii) governance structures and responsibilities related to skills monitoring; (iv) financial and resource implications of skill management; and (v) perceived risks and value of implementing a structured skills-monitoring system. Probing questions were used to elicit concrete examples and to triangulate perspectives across functional domains within each hospital.

### 3.4. Data Analysis

Data were analysed using reflexive thematic analysis following the six-phase framework proposed by Braun and Clarke [68,69]. This approach was selected because it is well suited for exploring complex organisational phenomena, enabling the identification of patterns of meaning across heterogeneous stakeholder perspectives while allowing for interpretive depth.

The analysis followed a primarily inductive orientation, allowing themes to emerge from the data, while being sensitised by the study’s conceptual focus on skills-based human capital management and workforce governance. This combination of inductive openness and theoretically informed interpretation allowed the analysis to remain grounded in empirical data while engaging with relevant conceptual frameworks.

Initial coding and theme development were conducted manually using a spreadsheet-based coding matrix. Given the relatively small dataset and the interpretive nature of reflexive thematic analysis, no dedicated qualitative software was used.

The first author (E.G.) coded all transcripts, while a second author (A.S.) independently coded a 25% sub-sample to support analytical credibility. Differences in coding were resolved through discussion and the use of analytical memos rather than calculation of inter-rater reliability coefficients, in line with reflexive thematic analysis principles [69].

The analytical process followed six stages: (1) familiarisation with the data; (2) initial coding without a predefined coding framework; (3) generation of candidate themes by grouping related codes; (4) iterative review and refinement of themes within and across institutions; (5) definition and naming of final themes; (6) interpretation and reporting.

Given the predefined organisational perspectives (HR, education, quality, and finance) and the inclusion of all Latvian university hospitals, the study prioritised informational adequacy and thematic sufficiency rather than statistical representativeness or universal saturation across all possible stakeholder groups.

Reflexivity was actively incorporated throughout the research process. All authors are affiliated with Rīga Stradiņš University and have research interests in healthcare workforce development and medical education, which may have influenced the framing and interpretation of the data. To address this, the lead researcher maintained a reflexive journal throughout data collection and analysis, documenting assumptions, analytical decisions, and emerging interpretations. Regular discussions were held with co-authors who were not directly involved in coding to challenge interpretations and minimise potential bias, particularly in relation to organisational structures, governance practices, and the interpretation of skill-related processes.

### 3.5. Ethical Considerations

The study was conducted in accordance with the Declaration of Helsinki and was approved by the Research Ethics Committee of Rīga Stradiņš University (decision No. 2-PEK-4/512/2025, 18 February 2025). Personal data were processed in accordance with Regulation (EU) 2016/679 (General Data Protection Regulation, GDPR). Informed consent was obtained from all participants prior to data collection. All quotations are presented in anonymised and aggregated form. Interviews were conducted in Latvian and translated into English by the authors with attention to conceptual equivalence. To ensure translation accuracy, back-translation by an independent translator was conducted for a 20% sub-sample of selected quotations.

## 4. Results

**Figure 1:**
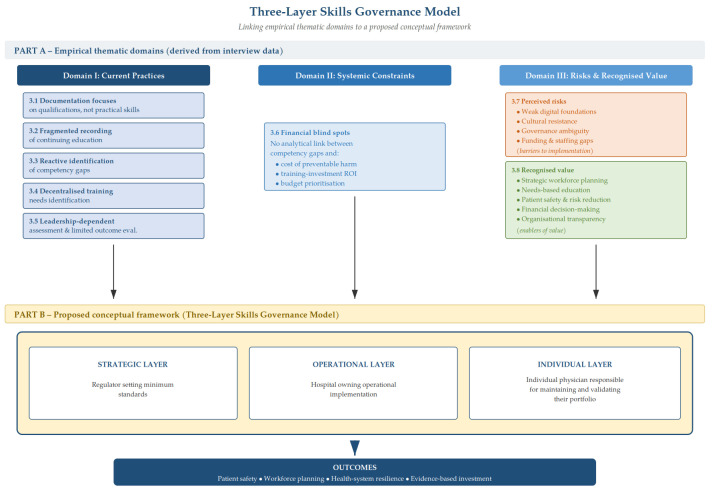
Data-informed conceptual synthesis of current skills documentation and assessment practices, systemic and financial constraints, and the perceived risks and value of skills monitoring, including the proposed Three-Layer Skills Governance Model.

Analysis of the interview data generated eight interrelated themes describing current practices, structural gaps, and perceived development needs in skills-based human capital management across Latvian university hospitals. Figure 1 depicts themes grouped into three analytical domains: (i) current documentation and assessment practices (Sections 3.1–3.5); (ii) systemic and financial constraints (Section 3.6); and (iii) perceived risks and recognised value of a structured skills-monitoring system (Sections 3.7 and 3.8).

These domains collectively reflect the organisational conditions under which physician skills are currently documented, interpreted, and used, as well as the barriers that limit their systematic integration into workforce governance.

### 4.1. Documentation Oriented Toward Qualifications Rather than Practical Skills

Across all institutions, documentation of physicians’ competencies was fragmented and primarily focused on formal qualifications rather than verifiable practical skills. Educational background, certification, and licensure were systematically recorded, particularly during recruitment and compliance-related processes. However, information on skills acquired through continuing professional development, internal training, or external courses was inconsistently captured and rarely integrated into organisational systems. None of the hospitals maintained a structured skills portfolio, competency matrix, or comparable mechanism for representing physicians’ practical capabilities at the organisational level. As a result, skills remained largely implicit and dispersed across individual records rather than being treated as organisationally visible and actionable resources.

*“At the moment, there is no structured list of skills. Information about seminars or courses is requested only if it is required for a specific position.”*

(HR, H1)

*“What really matters during recruitment are certificates. Beyond that, information on additional training is largely a matter of personal initiative.”*

(HR, H2)

*“We rely on certification and re-certification-there is no structured register of what specific procedures a doctor is able to perform.”*

(QA, H3)

This pattern indicates that competency documentation is largely oriented toward regulatory compliance rather than operational workforce management, limiting the organisation’s ability to assess and deploy skills strategically.

### 4.2. Fragmented Recording of Continuing Education

Processes for documenting continuing education were uneven across institutions and between training contexts. Activities organised internally—such as courses delivered by hospital education centres—were typically recorded, including attendance and credit accumulation. In contrast, externally acquired education remained largely outside organisational oversight and was often managed individually by physicians.

*“We do have records of what doctors complete within our training centre, but education acquired outside the hospital remains their personal responsibility.”*

(ED, H1)

*“That information exists, but it is not systematised. At the moment, we do not have a unified learning platform.”*

(ED, H2)

*“We document what we organise internally, but external courses remain in the doctor’s personal folder.”*

(HR, H3)

This dual system created a structural divide between formalised organisational knowledge and privately held professional development data. As a result, hospitals lacked a complete overview of workforce skills, which limits their ability to plan training, identify gaps, or align workforce capabilities with service needs.

### 4.3. Reactive Identification of Competency Gaps

The identification of competency and skill gaps was predominantly reactive rather than proactive. In most cases, gaps were recognised retrospectively—following adverse events, patient complaints, audit findings, or incident analyses—rather than through systematic or preventive assessment processes.

*“Usually, we identify skill gaps after something has gone wrong-through incidents or complaints.”*

(QA, H1)

*“Incident analysis is one of the main ways we understand whether the root cause was related to staff competencies or skills.”*

(QA, H2)

This reliance on retrospective detection suggests that skills are not systematically monitored as part of routine governance but instead become visible only when performance issues emerge. Consequently, opportunities for early intervention, prevention, and continuous improvement remain limited.

### 4.4. Training Needs Defined Through Decentralised Channels

Training needs were identified through multiple, decentralised channels, including immediate supervisors, audit findings, regulatory requirements, and individual initiative. Clinical-unit leaders played a central role in prioritising training, reflecting the highly localised nature of decision-making.

*“Most initiatives come from department heads. Then we also have audit findings and legal requirements that define mandatory training.”*

(ED, H3)

*“It is ultimately the responsibility of the direct manager-they know where gaps exist in their teams.”*

(HR, H1)

While this decentralised approach allowed flexibility and responsiveness to local needs, it also resulted in variability across departments and limited the development of a coordinated, organisation-wide approach to skill development. The absence of shared data further constrained cross-departmental alignment.

### 4.5. Leadership-Dependent Assessment and Limited Outcome Evaluation

Assessment of skills and competencies was largely dependent on the subjective judgement of direct supervisors or departmental leaders. Participants described reliance on day-to-day observation, informal knowledge of staff performance, and professional trust rather than on structured or standardised assessment tools.

*“The strongest indicator is the manager’s everyday observation-without portfolios or formal data. They know who performs which procedures.”*

(HR, H2)

*“Competence assessment depends on the manager-there are no common criteria.”*

(QA, H1)

This leadership-dependent model led to variability in assessment practices across departments and limited comparability of competence evaluations within organisations.

At the same time, evaluation of training outcomes was weakly developed. Training effectiveness was typically assessed through attendance records or participant feedback, with limited evidence of systematic outcome evaluation.

*“We evaluate training through surveys, but we don’t really have baseline indicators to measure impact over time.”*

(ED, H2)

*“If incidents decrease, then we can assume training helped-but this is not measured in a structured way.”*

(QA, H3)

The absence of baseline indicators and measurable outcomes made it difficult to assess whether training resulted in improved skills, reduced risk, or enhanced organisational performance. This further limits the integration of training into broader workforce governance processes.

### 4.6. Financial Blind Spots in Skills Investment

Across institutions, participants consistently reported that the financial implications of competency gaps were not systematically assessed or integrated into organisational decision-making processes. Although respondents acknowledged that insufficient skills may result in adverse events, inefficiencies, duplication of procedures, or extended treatment pathways, these consequences were rarely translated into measurable financial indicators or cost analyses.

*“We usually analyse incidents from a quality and safety perspective, but the financial consequences are not calculated in a structured way.”*

(FN, H1)

*“Training budgets exist, but we cannot determine which investments actually reduce costs later on, because we lack data connecting skills development with financial outcomes.”*

(FN, H2)

Investments in training and skills development were therefore weakly linked to financial planning and resource allocation. This separation suggests that physician skills are not currently treated as economic resources within organisational management systems, despite recognition of their impact on efficiency and patient outcomes.

From an organisational perspective, this creates a structural disconnect between workforce development activities and financial governance. Without mechanisms to quantify the relationship between skills, performance, and cost outcomes, decision-makers lack the evidence required to prioritise investments, assess return on training, or justify expenditures. As a result, skills development remains largely expense-driven rather than strategically aligned with organisational performance.

### 4.7. Perceived Risks of a Skills Monitoring System

Participants identified several interrelated risks associated with implementing a systematic skills-monitoring system. These risks spanned organisational, technological, cultural, and governance dimensions and highlighted potential constraints that could undermine implementation if not addressed. Rather than isolated barriers, these risks reflect the broader organisational readiness required for transitioning toward skills-based workforce governance.

#### 4.7.1. Weak Digital Foundations

Participants consistently emphasised the lack of robust digital infrastructure to support systematic skills tracking. Information related to competencies and training was distributed across multiple systems, spreadsheets, paper-based records, and training-centre databases, with limited interoperability.

*“At the moment, we do not have a learning platform-work is ongoing, but it is not yet in place.”*

(ED, H1)

*“Information on training completed outside the hospital is not captured in any system-it remains at departmental or individual level.”*

(HR, H2)

*“With five thousand employees, manual tracking is impossible.”*

(HR, H3)

Although some institutions were in the process of developing digital learning platforms, these systems were not yet fully integrated with HR, quality management, or operational decision-making processes. This fragmentation limits the ability to consolidate skill-related data and prevents the creation of a unified, organisation-wide view of workforce capabilities. As a result, digital systems currently support documentation of isolated activities rather than enabling integrated skills governance.

#### 4.7.2. Cultural Resistance

Cultural resistance emerged as a significant and multifaceted barrier. Participants reported concerns among physicians—particularly senior clinicians—regarding perceived threats to professional autonomy, fear of comparison or ranking, and reluctance to openly acknowledge skill gaps.

*“The biggest challenge is culture-accepting that skills monitoring should be part of everyday practice.”*

(ED, H3)

*“Senior doctors may perceive skills assessment as ranking or comparison, which raises ethical concerns and resistance.”*

(QA, H1)

*“If such systems are introduced without explanation, they may trigger fear, denial, and protection of personal boundaries rather than engagement.”*

(HR, H2)

These concerns indicate that skills monitoring is not interpreted as a neutral information system but as a potentially evaluative or controlling mechanism. Without clear communication, trust-building, and involvement of professionals in system design, implementation risks being perceived as externally imposed rather than as a supportive organisational tool.

#### 4.7.3. Governance Ambiguity

Participants highlighted ambiguity regarding responsibility for skills monitoring across multiple governance levels. While certification and re-certification are regulated nationally, additional competencies beyond formal requirements were perceived as falling between individual responsibility and organisational accountability.

*“Responsibility is shared between the individual professional, the hospital, and the state, but in practice there is no single owner of skills monitoring.”*

(HR, H1)

*“Certification defines the minimum requirement, but beyond that there is no clear framework specifying who should oversee or validate additional skills.”*

(QA, H2)

This distributed responsibility creates gaps in oversight and limits the development of coherent, organisation-wide approaches to skills management. In the absence of a clearly defined governance structure, skills monitoring remains fragmented and dependent on local initiatives rather than being embedded in institutional policy.

#### 4.7.4. Additional Funding and Staffing Requirements

Participants emphasised that developing and maintaining a skills-monitoring system would require dedicated financial resources and staff capacity. Systematic data collection, validation, updating, and integration into decision-making processes were perceived as resource-intensive activities.

*“Funding is needed for system development and maintenance, and for the workforce to operate it.”*

(FN, H2)

Without clearly allocated roles, funding mechanisms, and operational frameworks, skills monitoring risks being perceived as an additional administrative burden rather than as a strategic investment.

### 4.8. Recognised Value of a Skills-Monitoring System

Despite these risks, all respondents recognised the potential value of a structured skills-monitoring system. Participants described how improved visibility of physician skills could support organisational decision-making, improve efficiency, and enhance patient safety. Importantly, perceptions of value were consistent across functional domains, suggesting a shared recognition of the importance of skills as organisational resources.

#### 4.8.1. Strategic Workforce Planning

Participants emphasised that systematic visibility of physician skills would enable more strategic workforce planning. A structured overview of competencies was seen as essential for staff allocation, service organisation, and future workforce development.

*“If we knew exactly which competencies are available, we could plan services and staff allocation much more strategically.”*

(HR, H1)

In the absence of such information, planning decisions were described as reactive, experience-based, and dependent on informal knowledge rather than systematic data.

#### 4.8.2. Targeted and Needs-Based Education

Structured skill data were seen as critical for shifting from general or compliance-driven training toward targeted and needs-based education. Participants noted that clearer identification of skill gaps would allow more efficient allocation of training resources.

*“With a clear understanding of skill gaps, education could be planned purposefully, not just based on available courses.”*

(ED, H2)

This highlights the potential of skills monitoring to improve not only training relevance but also training efficiency and effectiveness.

#### 4.8.3. Patient Safety, Physician Safety and Risk Reduction

Participants consistently linked skills monitoring to improvements in patient safety, and also physician safety. Earlier identification of skill gaps was seen as enabling preventive action and reducing the likelihood of adverse events.

*“If skill gaps were visible earlier, some incidents could potentially be prevented.”*

(QA, H3)

Skills data were therefore perceived as a missing link connecting workforce development with quality and safety systems, allowing organisations to move from reactive incident analysis toward proactive risk management.

#### 4.8.4. Data-Informed Financial and Resource Decisions

Participants indicated that a skills-monitoring system could support more informed financial decision-making. By linking training investments to clearly identified needs, organisations could better justify expenditures and prioritise resources.

*“It would help justify why certain training investments are necessary and where funding should be directed.”*

(FN, H3)

Such integration would enable a shift from budget-driven decision-making toward evidence-based resource allocation.

#### 4.8.5. Organisational Transparency and Accountability

Skills visibility was also associated with increased organisational transparency and clearer accountability structures. Participants noted that a shared framework for documenting skills could support more consistent decision-making and clearer expectations.

*“A transparent overview would help everyone understand expectations-not to control individuals, but to support the organisation.”*

(HR, H2)

*“If we had a clear overview of competencies, we could plan education, staffing, and services in a much more strategic way.”*

(QA, H1)

At the same time, respondents emphasised that successful implementation would require a gradual, context-sensitive approach.

*“It would have to be implemented step by step, as a project with clearly defined phases.”*

(HR, H3)

*“Such a system should not be introduced as a control mechanism-it needs to start small and expand gradually as the organisation is ready.”*

(ED, H1)

These findings indicate that the perceived value of skills monitoring is conditional on its alignment with organisational culture, governance structures, and existing systems.

## 5. Discussion

### 5.1. Interpretation of Findings

To our knowledge, this study is among the first multi-institutional qualitative analyses to examine organisational approaches to physician skill governance across all Latvian university hospitals. The findings suggest that current organisational practices remain fragmented, reactive, and highly dependent on local managerial judgement rather than on unified systems of skill visibility and use. Although formal structures for certification, licensure, and continuing professional development are in place, none of the participating institutions maintained a comprehensive organisational overview of physicians’ practical skills. This limits the capacity for proactive workforce governance, targeted capability development, and data-informed decision-making.

A central finding of the study is that physician skills are not absent from organisational practice but insufficiently rendered visible as an organisational resource. Skills were reported to exist in multiple forms—through individual training histories, local managerial knowledge, certification records, and incident-based learning—but they were rarely consolidated into a structured system that could support planning, safety management, or resource allocation. From a skills-based human capital perspective, this indicates that an important organisational asset remains under-documented and therefore underutilised. The findings reinforce the argument developed in the Introduction: the governance challenge does not lie in whether physicians possess relevant skills, but in whether healthcare organisations can systematically identify, interpret, and mobilise those skills within operational and strategic processes.

The study also supports the analytical distinction between competencies, professional capability, and Entrustable Professional Activities (EPAs). Current organisational practices are largely oriented toward documentation of qualifications and certification, which function as proxies for competence but provide limited insight into practical skill execution or context-specific capability. Participants consistently described a gap between what is formally recorded and what can be inferred about day-to-day clinical performance. This highlights the limited translation of capability- and EPA-oriented approaches into organisational governance. While these frameworks offer conceptual tools for linking professional standards with practice, the findings suggest that they are not yet operationalised within hospital-level systems.

Another key contribution concerns the fragmentation of skills-related information across organisational domains. Human resources, education, quality, and finance functions each held partial views of physician skills, but these were not integrated into a shared governance mechanism. As a result, skills remain distributed across institutional silos rather than functioning as a collective managerial resource. This fragmentation has practical consequences: workforce planning depends on informal knowledge, training outcomes are rarely evaluated systematically, patient-safety learning is predominantly retrospective, and financial implications of skill gaps remain weakly articulated.

The “financial blind spots” identified in this study are particularly significant. Although participants recognised that skill deficits may contribute to inefficiencies, adverse events, and repeated interventions, these effects were rarely translated into measurable financial indicators. This suggests that the economic value of physician skills remains implicit rather than analytically integrated into decision-making. Consequently, investments in training and skill development are weakly connected to strategic financial planning, limiting the ability to assess return on investment or prioritise interventions based on expected organisational impact.

The findings also demonstrate that the challenges of skills monitoring are not purely technical. Weak digital infrastructure, governance ambiguity, cultural resistance, and resource constraints indicate that implementation should be understood as a complex organisational change process. In particular, concerns about professional autonomy and evaluation suggest that skill monitoring may be resisted if framed as a control mechanism. This aligns with broader evidence on assessment in professional contexts, where systems are more likely to be accepted when positioned as developmental and supportive rather than punitive [70,71,72].

At the same time, there was strong convergence across stakeholder groups regarding the potential value of improved skill visibility. Human resources participants linked skill data to workforce planning; education stakeholders emphasised its role in targeted learning; quality professionals connected it to risk identification; and finance participants highlighted its relevance for more evidence-based investment decisions. This consistency suggests that the conceptual importance of skills as organisational resources is widely recognised. The main constraints lie in organisational structures, integration mechanisms, and supporting infrastructure rather than in conceptual disagreement.

### 5.2. From Thematic Findings to a Data-Informed Conceptual Framework

Building on the empirical findings, we propose the Three-Layer Skills Governance Model as a data-informed conceptual framework for interpreting how physician skill information may be governed across small health systems. The model is not presented as a directly validated empirical structure or as a formally derived theory. Rather, it represents a practice-oriented conceptual synthesis that organises the thematic findings in light of the study’s theoretical framing.

The rationale for distinguishing three layers arises from the recurring way participants described skill-related information as being shaped at different but interconnected levels. First, participants referred to external and regulatory expectations that define minimum professional standards. Second, they described hospital-level processes through which skill-related information is documented, interpreted, and used in management practices. Third, they highlighted the continued role of individual physicians in maintaining records of acquired skills, particularly those obtained outside organisational systems. The model therefore does not assume the existence of a formalised governance architecture but instead conceptualises how physician skill governance may be understood as a multi-level phenomenon.

At the strategic layer, the model refers to the regulatory and professional environment in which baseline requirements for physician EPAs, competencies, and skills are established. This includes licensure systems, certification processes, and externally defined professional standards. The findings indicate that these structures are currently the most formalised and visible components of skills documentation, but they primarily reflect minimum qualification, while visible and verifiable components of practical skill execution remain insufficiently captured.

At the operational layer, the model focuses on the hospital as an organisational site where skill-related information may be documented, interpreted, and applied. The findings demonstrate that this level is characterised by fragmentation, with different organisational functions maintaining separate and only partially connected data systems. The operational layer therefore represents the organisational processes required to transform dispersed information into actionable knowledge that can support workforce planning, education, quality management, and financial decision-making, including the development of an integrated institutional skills portfolio.

At the individual layer, the model reflects the continued role of physicians in maintaining their own records of training, skills, competencies, and professional development. Participants described how externally acquired skills, courses, and certifications are often documented individually rather than integrated into organisational systems. This layer highlights the importance of individual-level data as a component of skills governance, while also illustrating its current lack of integration into institutional processes, and the need for physicians to actively maintain their own individual skills portfolios.

Taken together, the model provides a structured way of interpreting how physician skills governance may be conceptualised across regulatory, organisational, and individual dimensions. It does not replace existing competency frameworks but complements them by focusing on how skill-related information is captured, connected, and used within organisational systems. Its primary contribution lies in offering a conceptual lens through which fragmented organisational practices may be understood and potentially aligned.

### 5.3. Practical Implications for Skills-Based Human Capital Management

The practical implications of the findings lie in clarifying the organisational conditions required for developing more systematic approaches to skills-based human capital management. Rather than suggesting immediate full-scale implementation, the findings indicate that such approaches should begin with a structured assessment of the current situation. This involves identifying existing data sources, understanding how information is currently used, and mapping gaps and inconsistencies across organisational processes.

The findings also suggest that implementation is likely to be more effective if approached incrementally. Hospitals may begin by focusing on selected areas where the relevance of skill visibility is most evident, such as high-risk clinical domains or training-intensive environments. This would allow organisations to test data structures, governance responsibilities, and user acceptance in a controlled and contextually meaningful way.

Importantly, the results indicate that skills monitoring should not be approached solely as a technical or digital solution. While information systems are necessary, they are insufficient without corresponding governance arrangements, clearly defined responsibilities, and organisational trust. Skill-related data must be embedded within decision-making processes and interpreted within shared frameworks that are accepted by both managerial and clinical stakeholders.

Within this context, the proposed Three-Layer Skills Governance Model may serve as a heuristic framework for supporting organisational reflection and planning. It highlights the need to align regulatory standards, hospital-level processes, and individual skill documentation in order to create a more coherent approach to skills-based workforce governance. Its value lies not in prescribing a fixed structure, but in supporting institutions in developing context-appropriate solutions. In this sense, the findings can be interpreted through a skills-based human capital perspective, where the organisational value of physician skills depends not only on their presence, but on their visibility, structuring, and integration into governance processes. From both resource-based and systems perspectives, physician skills function as strategic but underutilised resources, whose contribution to organisational performance emerges only when they are systematically embedded within data infrastructures and decision-making routines.

### 5.4. Strengths and Limitations

This study has several strengths. It included all Latvian university hospitals and incorporated multiple organisational perspectives that are directly involved in skills governance: human resources, education, quality, and finance. This multi-functional approach provides insight into how skills-related information is distributed across organisational domains and highlights the mechanisms through which fragmentation emerges. The use of reflexive thematic analysis, combined with structured reporting and explicit reflexivity, supports the transparency and credibility of the findings.

Several limitations should also be acknowledged. First, the qualitative design focuses on organisational perspectives rather than measurable outcomes and does not aim for statistical generalisation. Second, although all university hospitals were included, the sample size was limited and focused on selected organisational roles. Third, the study did not include practising physicians or clinical department leaders, whose perspectives would be important for understanding the professional and experiential dimensions of skills assessment and governance. Fourth, the proposed conceptual model is not empirically validated but represents a theoretically informed interpretation of the findings. Finally, the study did not directly quantify the financial impact of skill gaps, indicating the need for further work in this area.

### 5.5. Future Research

The findings suggest several directions for future research. Further studies could extend the analysis to other healthcare settings, including regional and private institutions, to assess the transferability of the findings. Qualitative research involving clinicians and trainees would provide complementary perspectives on skill documentation and assessment. Implementation studies are needed to explore the feasibility and acceptability of structured approaches to skills monitoring in specific clinical contexts.

In addition, the financial dimension identified in this study warrants further investigation. Future research could examine the relationship between skill-related data and outcomes such as incident rates, efficiency indicators, or cost structures. Comparative studies across different health systems may also help identify context-specific and generalisable elements of skills-based workforce governance.

## 6. Conclusions

This study examined how information on physician skills is organisationally documented, interpreted, and used for workforce governance across Latvian university hospitals. The findings indicate that current practices remain fragmented, predominantly qualification-oriented, and weakly integrated across organisational domains. Although skill-related information exists in multiple forms—including certification records, local managerial knowledge, training documentation, and individual professional portfolios—it is rarely consolidated into a structured, organisation-wide system that would enable its systematic use for workforce planning, education, quality management, and financial decision-making.

A central conclusion of this study is that physician skills are widely recognised as important but are not yet systematically rendered visible as an organisational resource. The main constraints identified by participants relate not to conceptual disagreement about the role of skills, but to organisational and infrastructural factors, including fragmented data systems, unclear governance responsibilities, limited digital integration, and cultural concerns regarding assessment and professional autonomy. These findings suggest that the key challenge lies in developing organisational mechanisms capable of making skills visible, interpretable, and actionable across multiple functional domains.

These results reinforce the theoretical argument developed in the literature review: within a skills-based human capital framework, the value of human capital does not reside solely in the possession of competencies, but in the organisation’s ability to identify, structure, and mobilise skills as actionable resources. From a resource-based perspective, physician skills constitute strategically relevant intangible assets, whose contribution to organisational performance depends on their integration into governance structures and decision-making processes. A systems perspective further highlights that the organisational value of skills emerges only when skill-related data are embedded within interconnected processes of assessment, learning, deployment, and coordination.

The study contributes to the literature by providing empirical insight into organisational approaches to skills governance in a small, centralised health system context. Building on these findings, the Three-Layer Skills Governance Model is proposed as a data-informed conceptual framework for interpreting how physician skill information may be aligned across strategic, operational, and individual levels. The model is not intended as a validated governance structure, but as an interpretive and practice-oriented synthesis that highlights the need to connect regulatory standards, organisational processes, and physician-held skill records within a coherent governance architecture.

From a practical perspective, the findings suggest that the development of skills-based human capital management approaches should begin with systematic assessment of existing practices, data sources, and organisational processes, rather than immediate large-scale system design. Incremental and context-sensitive implementation strategies—particularly those aligned with existing workflows, clinical priorities, and organisational culture—may provide a more feasible pathway for improving skills visibility and integration.

Overall, improving the visibility and usability of physician skill information represents an important opportunity for strengthening workforce governance in healthcare organisations. In small health systems characterised by limited capacity and concentrated expertise, such developments may support more strategic workforce planning, more targeted capability development, and more effective alignment between human capital and organisational performance.

## Data Availability

The interview transcripts generated and analysed in this study are not publicly available due to participant confidentiality and the conditions of the informed consent obtained.

## Abbreviations

The following abbreviations are used in this manuscript:

API	Application Programming Interface
CanMEDS	Canadian Medical Education Directions for Specialists
COREQ	Consolidated Criteria for Reporting Qualitative Research
EPA	Entrustable Professional Activity
EU	European Union
GDP	Gross Domestic Product
GDPR	General Data Protection Regulation
HCM	Human Capital Management
HR	Human Resources
HRH	Human Resources for Health
HRM	Human Resource Management
IT	Information Technology
OECD	Organisation for Economic Co-operation and Development
RBV	Resource-Based View
SRQR	Standards for Reporting Qualitative Research
WHO	World Health Organization
